# Prognostic value of CASC15 and LINC01600 as competitive endogenous RNAs in lung adenocarcinoma: An observational study

**DOI:** 10.1097/MD.0000000000036026

**Published:** 2023-11-10

**Authors:** Fangbin Zhou

**Affiliations:** a Department of Tropical Diseases, Naval Medical University, Shanghai, China.

**Keywords:** CASC15, competitive endogenous RNAs, LINC01600, long noncoding RNAs, lung adenocarcinoma, prognosis

## Abstract

Long noncoding RNAs (lncRNAs) can directly or indirectly regulate gene expression through interacting with microRNAs (miRNAs). Competitive endogenous RNAs render the roles of lncRNAs more complicated in the process of tumor occurrence and progression. However, the prognostic value of lncRNAs as potential biomarkers and their functional roles as competitive endogenous RNAs have not been clearly described for lung adenocarcinoma (LUAD). In the present study, the aberrant expression profiles of lncRNAs and miRNAs were analyzed at cBioPortal by interrogating LUAD dataset from The Cancer Genome Atlas (TCGA) database with 517 tissue samples. A total of 92 lncRNAs and 125 miRNAs with highly genetic alterations were identified. Further bioinformatics analysis was performed to construct a LUAD-related lncRNA-miRNA-mRNA ceRNA network, which included 24 highly altered lncRNAs, 21 miRNAs and 142 mRNAs. Some key lncRNAs in this network were subsequently identified as LUAD prognosis-related, and of those, CASC15 and LINC01600 both performed the potential prognostic characteristics with LUAD regarding OS and recurrence. Comprehensive analysis indicated that the expression of LINC01600 was significantly associated with KRAS mutation and lymph node metastasis, and CASC15 and LINC01600 were significantly tended towards co-occurrence, which may be due to the similarity of genes co-expressed by these 2 lncRNAs. Our findings provided novel insight into better understanding of ceRNA regulatory mechanisms in the pathogenesis of LUAD and facilitated the identification of potential biomarkers for prognosis.

## 1. Introduction

Lung cancer is the most frequently reported malignancy worldwide, leading to more than 2.21 million incidences and almost 1.80 million deaths, as estimated in 2020.^[1]^ Lung adenocarcinoma (LUAD) is the most common histological subtype of lung cancer, which accounts for an approximate proportion of 40%. There is a huge number of pivotal molecules like tyrosine kinase inhibitors identified for targeted therapies, which have dramatically improved treatment for LUAD patients.^[2–5]^ However, the overall 5-year survival rate of LUAD patients has not been significantly improved over the past few decades. Therefore, it is urgently required to understand the molecular nature of LUAD pathogenesis and develop new targets for the diagnosis and treatment of LUAD.

Increasing evidence has emphasized the involvement of noncoding RNAs (ncRNAs) in the process of tumor occurrence and progression.^[6,7]^ ncRNAs are a class of RNA molecules without protein-coding function, and microRNAs (miRNAs) and long noncoding RNAs (lncRNAs) are 2 typical subtypes. miRNAs can repress the expression of targeted mRNAs by partial complementation binding to the mRNA sequences, which was also named miRNA response elements (MREs).^[8]^ It has been well-documented that miRNA-mRNA interactions involve a variety of biological processes (BP), including carcinogenesis and tumor metastasis.^[9,10]^ In contrast, lncRNAs function via more multiple mechanisms.^[11]^ In addition to directly targeting mRNAs, lncRNAs can function as competing endogenous RNAs (ceRNAs) to indirectly regulate mRNAs through competing for shared miRNAs. In 2011, ceRNA hypothesis was proposed by Salmena and colleagues, which states that lncRNAs, mRNAs and other RNAs can act as natural miRNA sponges to inhibit miRNA function by competitive binding to shared MREs.^[12]^ Later, this hypothesis was updated and validated experimentally by more and more convincing evidences.^[13–15]^ Pandolfi et al demonstrated the existence of phosphatase and tensin homologue and its ceRNAs interaction among mRNAs in vitro and in vivo.^[16]^ Kallen et al found that H19 can affect the expression of endogenous let-7 targets through acting as a molecular sponge to sequester let-7.^[17]^ The above investigations regarding the lncRNA-miRNA-mRNA ceRNA networks provide a better understanding of the roles of lncRNA-miRNA interactions in mRNAs regulation and tumorigenesis.

With the development of multicentered cancer genomic projects, such as The Cancer Genome Atlas (TCGA), Gene Expression Omnibus and the Wellcome Trust Sanger Institute’s Cancer Genome Project, bioinformatic technologies and tools for high throughput genome analysis were increasingly created and more and more research focused on mining database to study ceRNA networks in cancer. Klonowska et al summarized a highly illustrated review of the common 7 web-based portals for the interpretation of the data.^[18]^ All of the selected portals were user-friendly and expected to contribute to a better understanding of cancer molecular etiology and the translation of genomic knowledge into clinical practice. In addition, to annotate the associations between ncRNAs and cancers, some disease-associated algorithms and databases, such as StarBase, miRTarBase, and DIANA-LncBase/TarBase were developed.^[19–22]^ The integration of web portals and datasets made it feasible to construct a ceRNA regulatory network in cancer. Recently, based on TCGA or Gene Expression Omnibus data analysis, some lncRNA-miRNA-mRNA ceRNA regulatory networks were constructed among gastric, breast, and bladder cancer, and numerous prognosis-related ncRNA biomarkers were identified.^[23–25]^ A few studies also used different sample datasets to investigate the LUAD related lncRNA-miRNA-mRNA ceRNA networks and identify several prognostic lncRNAs.^[26,27]^ However, comprehensive analysis of ncRNAs related to LUAD at genome-wide RNA profiles and large sample scale were limited and the relationship between cancer specifc lncRNAs and clinical features still remained to be elucidated.

In this study, we interrogated the LUAD dataset of TCGA containing 517 samples at cBioPortal with the latest updated lncRNAs and miRNAs database from The HUGO Gene Nomenclature Committee (HGNC) and constructed a regulated lncRNA-miRNA-mRNA based ceRNA networks. A total of 24 lncRNAs, 19 miRNAs and 142 mRNAs were involved in this network and several lncRNAs were identified as LUAD prognosis-related. Furthermore, CASC15 and LINC01600 were proven to perform the potential prognostic characteristics with LUAD regarding both overall survival (OS) and recurrence and were further disclosed the correlation with the clinical features and mutual exclusivity status.

## 2. Materials and Methods

### 2.1. cBioPortal and LUAD TCGA dataset

The cBioPortal for Cancer Genomics (http://www.cbioportal.org) is an open-access resource, which provides visualization, analysis tools for more than 5000 tumor samples from 105 cancer studies in TCGA pipeline. The customized search interface enabled researchers to interactively and individually explore genetic alterations across samples from other cancer studies and specific genes. A total of 586 LUAD tissue samples were retrieved from Lung Adenocarcinoma (TCGA, Provisional) dataset. In this study, Samples without completed data (including age, gender, race, TNM stage, and histological subtype and grade) for analysis or not histologically diagnosed as LUAD were excluded and a total of 517 LUAD tissue samples were obtained. This study is in accordance with the publication guidelines provided by TCGA (https://cancergenome.nih.gov/publications/publicationguidelines). As the data comes from the TCGA database, further approval by the Ethics Committee was not required.

### 2.2. Identification of lncRNAs and miRNAs with genetic alterations

Although there are increasing numbers of human lncRNAs and miRNAs reported from databases, the nomenclature of lncRNAs and miRNAs is still disordered. In this study, a total of 3885 lncRNAs and 1878 miRNAs were downloaded from HGNC (http://www.genenames.org/) for our analysis (Tables S1, Supplemental Digital Content, http://links.lww.com/MD/K665 and S2 Supplemental Digital Content, http://links.lww.com/MD/K666). All lncRNAs and mRNAs were input and recognized in the cBioPortal, and those of which were not included in this database were excluded. Alterations in the somatic mutations, DNA copy number alterations (CNAs) from GISTIC, mRNA, and microRNA expression were analyzed and calculated based on the clinical data from cBioPortal.

### 2.3. lncRNA–miRNA interaction analysis

The miRNAs targeted by highly altered lncRNAs were predicted using starBase (version 2.0, http://starbase.sysu.edu.cn/) and DIANA-LncBase (Experimental v.2, https://dianalab.e-ce.uth.gr/html/diana/web/index.phpr=tarbasev8%2Fdownloaddataform.) databases. Results from these 2 databases were combined and intersected with the above highly altered miRNAs. The intersection contained highly altered miRNAs were targeted by highly altered lncRNAs. At last, several LUAD-related lncRNA-miRNA pairs were thus obtained.

### 2.4. Prediction of mRNAs targeted by miRNAs and construction of a LUAD-related lncRNA–miRNA–mRNA ceRNA network

mRNAs targeted by miRNAs were retrieved from miRTarBase (version 6.0, https://mirtarbase.cuhk.edu.cn/~miRTarBase/miRTarBase_2022/php/index.php.) and DIANA-TarBase (version 8.0, http://carolina.imis.athena-innovation.gr/diana_tools/web/index.php?r=tarbasev8%2Findex). miRTarBase is a database that has accumulated more than three hundred and sixty thousand miRNA-target interactions (MTIs), which are collected by manually surveying pertinent literature and mining the text systematically to filter research articles related to functional studies of miRNAs. DIANA-TarBase was a database with experimentally supported miRNA-mRNA interations. In this part, mRNA targets were predicted via experimental module and had a high Pr. score. miRNAs with <5% genetic alteration screened via cBiPortal were excluded, and those remaining miRNAs targeted by highly altered miRNAs were extracted and then integrated with differentially expressed miRNA–mRNA regulatory relationships, generating a LUAD-related miRNA-mRNA regulation network.

The above lncRNA-miRNA and miRNA-mRNA regulatory networks were combined to obtain a comprehensive lncRNAmiRNA-mRNA ceRNA regulatory network. The network graph was constructed and visualized using Cytoscape v3.0 (http://www.cytoscape.org/). Cytoscape is an open source software platform for visualizing molecular interaction networks and biological pathways and integrating these networks with annotations, gene expression profiles and other state data.

### 2.5. Functional and pathway annotation of mRNAs in the ceRNA network

In order to reveal LUAD-related biological functions and pathways, genes involved in ceRNA regulatory network were input into the Database for Annotation, Visualization and Integrated Discovery for GO (gene ontology) BP analysis and KEGG (Kyoto Encyclopedia of Genes and Genomes) pathway analysis. Fisher exact test was used during the enrichment process. At last, STRING software was used to visually map the PPI network of targeted genes.

### 2.6. Identification of prognosis-associated lncRNAs

The expression value of each lncRNAs in the ceRNA network and the survival information of each sample were extracted from TCGA dataset. The cBioPortal generated the Kaplan–Meier curve following input of the gene name for disease or progression-free-survival. *P* < .05 was considered to indicate a statistically significant difference.

## 3. Results

### 3.1. Genetic alteration profiles of lncRNAs and miRNAs in LUAD

To draw the genetic alteration profiles of lncRNAs in LUAD, A total of 3855 lncRNAs downloaded from HGNC were selected and performed a primary search in cBioPortal (Table S1, Supplemental Digital Content, http://links.lww.com/MD/K665). The results indicated that the profile of lncRNAs genetic alterations was heterogeneous and divided into 3 groups based on the altered frequencies (Table S3, Supplemental Digital Content, http://links.lww.com/MD/K667). The first, and smallest group, which comprises roughly 0.18% (7/3885) of the lncRNAs database, includes lncRNAs that are highly variable and had alterations in 10% cases or above. A second, larger group includes additional, frequently variable lncRNAs that had alterations more than 5% but <10% cases (2.19%, 85/3885). The above 92 lncRNAs defined highly altered lncRNAs in this study (Table S3, Supplemental Digital Content, http://links.lww.com/MD/K667). The third group contains lncRNAs that were rarely variable (<5%) or no alterations (97.61%, 3792/3885).

The genetic alteration profile of 1878 miRNAs was also divided into 3 groups as Table S2, Supplemental Digital Content, http://links.lww.com/MD/K666 shown. miRNAs that had alterations in 10% cases or above accounted for 1.28% (24/1878) and alterations more than 5% but <10% cases accounted for 5.38% (101/1878). The above 125 miRNAs defined highly altered miRNAs (Table S4, Supplemental Digital Content, http://links.lww.com/MD/K668). The remaining 93.34% (1753/1878) stood for the rare cases (<5%) or no alterations.

### 3.2. LncRNA-miRNA interaction network predicted by StarBase and DIANA-LncBase

Although noncoding RNAs play vitally physiological roles in the initiation and progression of LUAD, the precise mechanism and the regulation network of lncRNAs and miRNAs remain to be elucidated. Since miRNAs are interacting with lncRNAs through MREs within ceRNA network, the first thing we should do was to screen the potential MREs in lncRNAs. The database of miRNA–lncRNA interactions was downloaded from StarBase, which was performed to screen the potential MREs. One hundred twenty-five highly altered miRNAs were selected to be interrogated whether these miRNAs are targeted on 92 highly altered lncRNAs. The results showed that 36 of 92 lncRNAs were included in this database and 21 of 125 miRNAs may interact with 24 of 36 lncRNAs. Finally, a total of 54 lncRNA-miRNA pairs were obtained (Table 1).

**Table 1:** Putative miRNAs targeting lncRNA.

lncRNAs	miRNAs
CASC15	hsa-mir-298
DANCR	hsa-mir-135b
DLEU2	hsa-mir-30b, hsa-mir-30d
HCG18	hsa-mir-214, hsa-mir-296, hsa-mir-148a, hsa-mir-320a, hsa-mir-30b, hsa-mir-30d
HCP5	hsa-mir-214, hsa-mir-29c
HOTAIR	hsa-mir-214, hsa-mir-148a
JPX	hsa-mir-708
LINC00174	hsa-mir-148a, hsa-mir-31
LINC00242	hsa-mir-214
LINC00339	hsa-mir-214, hsa-mir-148a
LINC00467	hsa-mir-4735
LINC00652	hsa-mir-214
LINC00662	hsa-mir-190b
LINC00667	hsa-mir-148a, hsa-mir-31
LINC01600	hsa-mir-298
LINC00696	hsa-miR-1273a, hsa-miR-4756
MALAT1	hsa-mir-92b, hsa-mir-135b, hsa-mir-708, hsa-mir-383, hsa-mir-320a, hsa-mir-30b, hsa-mir-30d, hsa-mir-491, hsa-mir-298
MIAT	hsa-mir-29c
NEAT1	hsa-mir-214, hsa-mir-708, hsa-mir-4458, hsa-mir-339, hsa-mir-383, hsa-mir-320a
PVT1	hsa-mir-190b, hsa-mir-488
SNHG1	hsa-mir-4735, hsa-mir-383
SNHG12	hsa-mir-320a
SNHG15	hsa-mir-4735
TUG1	hsa-mir-29c, hsa-mir-31

### 3.3. mRNAs targeted by miRNAs

To construct the lncRNA–miRNA–mRNA ceRNA regulatory network, mRNA targeted by miRNAs should be searched. miRTarBase and DIANA-LncBase were used to investigate it. For miRTarBase, each miRNA–mRNA pair was experimentally validated by at least 3 of the following methods, including reporter assay, Western blot, qPCR or NGS. For DIANA-LncBase, mRNA targets were predicted via experimental module and had a high Pr. score. Finally, 163 mRNAs were targeted by 21 miRNAs and those with <5% genetic alteration screened via cBiPortal were excluded. Finally, 142 mRNAs were predicted as miRNA targets and a total of 162 miRNA-mRNA pairs were obtained (Table 2).

**Table 2:** Validated mRNAs’ targets from TarBase.

miRNAs	mRNAs
hsa-mir-135b	ARID1A, MTCH2, STAT6, BGLAP, APC, ACVR1B, EVI5, RUNX2, MAFB, PPP2R5C, SMAD5, MID1, BMPR2, THBS2
hsa-mir-148a	AKT2, USP4, ITGB8, ROCK1, DNMT3B, BCL2L11, CDC25B, CDKN1B, IRS1, SMAD2, TGIF2, ALCAM, CENPF, IKBKB, MAP3K9, RPS6KA5, STAT3, TGFB2, INO80, MAFB, VAV2
hsa-mir-190b	PTEN, BCL2
hsa-mir-214	MAPK14, RAB5B, UBE2I, FLOT1, HDGF, MAPK8, BIRC5, EZH2, KIF1B, TRPS1, BCL2L2, SRGAP1, SRGAP2, ARL2, BCL2L11, CDK3, CDK6, CPD, ING4, MAP2K3, PIM1, RAB15, FGFR1, JAG1, NRAS, PSMD10, RASSF5, TWF1, ARHGAP10, CAPN5, CPEB4, IGF1R, PAPPA, PTEN, QKI, TP53, TWIST1
hsa-mir-296	SCRIB, HGS, WNK4, IKBKE
hsa-mir-298	BACE1, ZFR, MATR3, APP
hsa-mir-29c	AKT2, DNMT3A, CREB5, MCL1, TDG, TFAP2C, DNMT3B, Mmp15, CDK6, RCC2, SIRT1, SRSF10, VEGFA, AKT3, FBN1, GAPDH, BACE1, BCL2, CDC42, COL15A1, CTNND1, PTEN
hsa-mir-30b	RORC, CCNE2, BCL6, SNAI1
hsa-mir-30d	EZH2, RUNX2, CASP3, SNAI1
hsa-mir-31	MET, NUMB, CREG1, RHOA, HOXC13, YY1, NFAT5, SATB2, RET, ARPC5, FZD3, HIF1AN, FOXP3, SELE, RDX, DMD, WASF3
hsa-mir-320a	ARF1, SUZ12, FOXM1, MCL1, RAB14, HOXA10, TRPC5, NFATC3, RUNX2, GNAI1, PTEN
hsa-mir-339	PTP4A1, BCL6, BACE1
hsa-mir-383	ATR, PPP1R10, PRPF31, VEGFA, IGF1R, PRDX3
hsa-miR-4458	NDC1, PXT1, IGF1R
hsa-miR-4735	ZNF507
hsa-mir-488	BCL2L11
hsa-mir-491	EGFR, CAPNS1, GIT1
hsa-mir-708	AKT2, EYA3, BIRC5, MPL
hsa-mir-92b	PRMT5, NLK
hsa-miR-1273a	SMG1
hsa-miR-4756	CCND1

### 3.4. Construction of a lncRNA–miRNA–mRNA ceRNA network in LUAD

**Figure 1.**
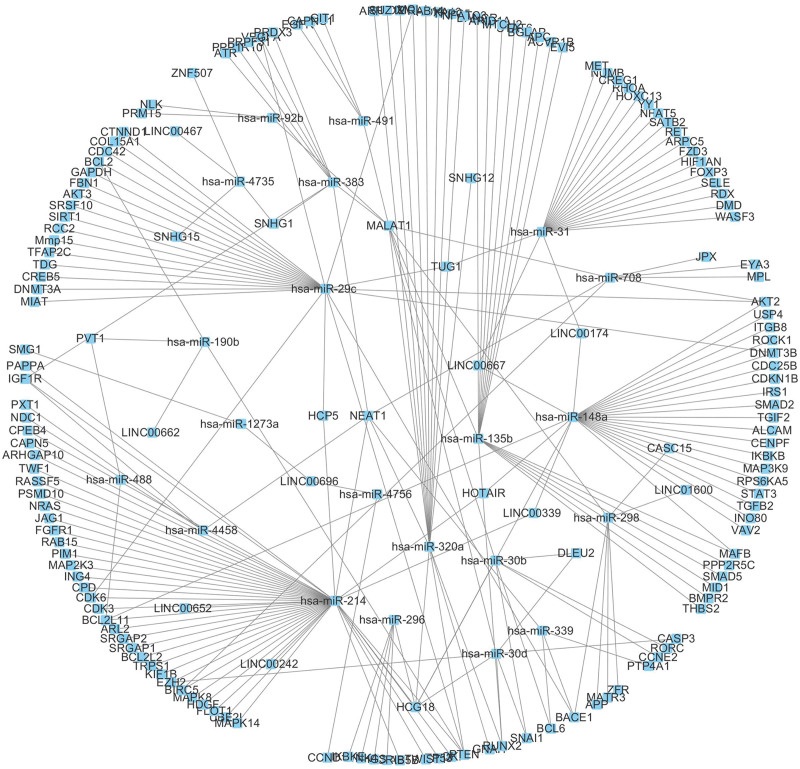
The lncRNA–miRNA–mRNA ceRNA network in LUAD. The network was constructed with Attribute Circle Layout algorithm and all lncRNAs and miRNAs were located into the circle.

To better understand the role of ncRNAs in LUAD and to further elucidate the interaction between these lncRNAs and miRNAs, a dysregulated lncRNA-miRNA-mRNA ceRNA network of LUAD was constructed. Cytoscape software was used to visualize the network. As shown in Figure 1, a total of 187 nodes and 217 edges were mapped and a total of 24 lncRNAs, 21miRNAs and 142 mRNAs were involved.

### 3.5. GO and KEEG pathway analysis

**Figure 2.**
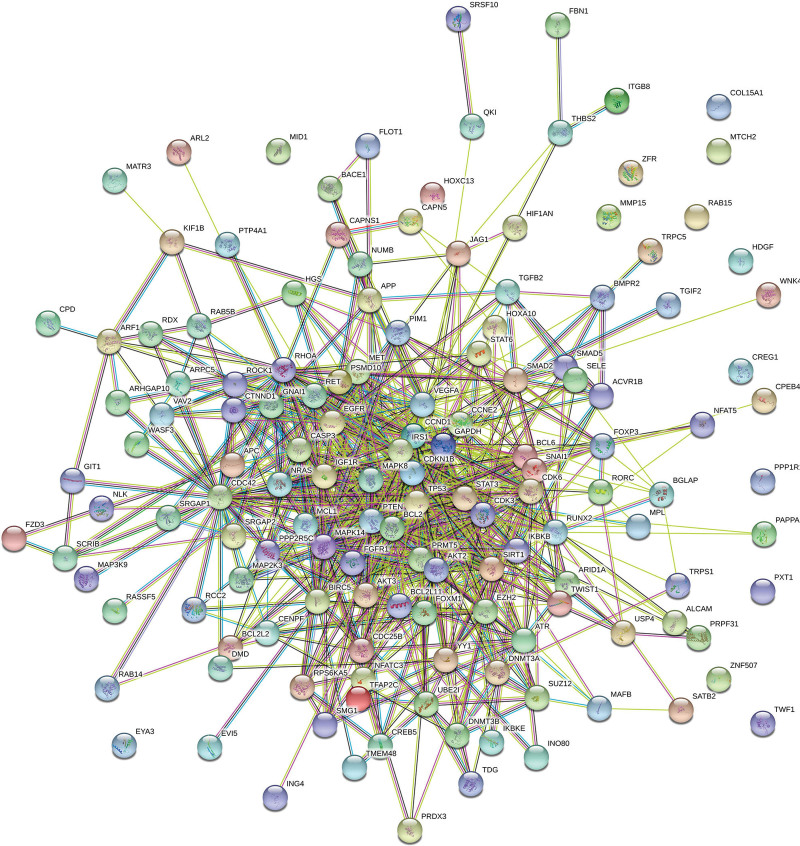
PPI network of mRNAs in the ceRNA network. 142 mRNA were submitted to the STRING database and visualized as a PPI network, which consisted of 142 nodes and 765 edges.

In order to investigate the functional processes involved in LUAD development and progression, mRNAs in the ceRNA network were subjected to Fisher exact test-based GO biological and functional process analysis. 142 mRNA were submitted to the STRING database and visualized as a PPI network, which consisted of 142 nodes and 765 edges (Fig. 2). The results of functional analysis of miRNAs were generated with DAVIAD. miRNAs were categorized into three functional groups: BP, molecular function and cellular component (CC). According to number of genes involved, we listed the top 10 significantly related GO analysis as Table 3 shown. The results indicated that in the BP, up-regulated genes were mainly enriched in negative regulation of transcription from RNA polymerase II promoter, negative regulation of cellular senescence, positive regulation of gene expression, negative regulation of apoptotic process and so on. In the CC analysis, the genes were mainly enriched in transcription factor complex nucleoplasm, PML body, cytoplasm, nuclear chromatin, focal adhesion, cytosol, nucleus, axon and extracellular exosome. For molecular function, up-regulated genes were significantly enriched in ATP binding, protein serine/threonine kinase activity, RNA polymerase II core promoter sequence-specific DNA binding and so on. The results demonstrated that most mRNAs in the ceRNA network were significantly enriched in biological or cellular process, cell components and kinase activities.

**Table 3:** Go analysis of mRNAs in the ceRNA network.

Term	Count	*P*-value*
*GO-BPs*		
GO:0000122~negative regulation of transcription from RNA polymerase II promoter	16	1.27E−06
GO:2000773~negative regulation of cellular senescence	4	4.04E−05
GO:0010628~positive regulation of gene expression	9	4.96E−05
GO:0043066~negative regulation of apoptotic process	11	7.82E−05
GO:0045944~positive regulation of transcription from RNA polymerase II promoter	15	2.72E−04
GO:0032000~positive regulation of fatty acid beta-oxidation	3	.00111
GO:0071479~cellular response to ionizing radiation	4	.002047
GO:0006978~DNA damage response, signal transduction by p53 class mediator resulting in transcription of p21 class mediator	3	.002299
GO:0001657~ureteric bud development	4	.002308
GO:0007283~spermatogenesis	7	.004424
*GO-CCs*		
GO:0005667~transcription factor complex	8	7.24E−05
GO:0005654~nucleoplasm	25	1.34E−04
GO:0016605~PML body	6	1.35E−04
GO:0005737~cytoplasm	34	2.10E−04
GO:0000790~nuclear chromatin	7	3.74E−04
GO:0005925~focal adhesion	10	9.86E−04
GO:0005829~cytosol	14	.007474
GO:0005634~nucleus	27	.008162
GO:0030424~axon	5	.008597
GO:0070062~extracellular exosome	27	.031307
*GO-MFs*		
GO:0005524~ATP binding	27	7.72E−05
GO:0004674~protein serine/threonine kinase activity	10	1.33E−04
GO:0000979~RNA polymerase II core promoter sequence-specific DNA binding	4	1.13E−02
GO:0004713~protein tyrosine kinase activity	3	1.98E−02
GO:0001077~transcriptional activator activity, RNA polymerase II core promoter proximal region sequence-specific binding	6	2.61E−02
GO:0003700~transcription factor activity, sequence-specific DNA binding	8	3.08E−02
GO:0008384~IkappaB kinase activity	2	.030858
GO:0003886~DNA (cytosine-5-)-methyltransferase activity	2	.030858
GO:0005525~GTP binding	8	.038338
GO:0008201~heparin binding	4	.043016

BP = biological process; CC = cellular component; MF = molecular function.

* *P* value < .05 was considered as threshold values of significant difference.

To understand the signal pathways involved in ceRNA network, KEEG pathway analysis was performed. The top 15 KEGG pathways in our study were listed as Table 4 shown. Ten cancer-related pathways were identified, including: MicroRNAs in cancer, pathways in cancer, proteoglycans in cancer, pancreatic cancer, prostate cancer, colorectal cancer, melanoma, central carbon metabolism in cancer, glioma, and endometrial cancer. Five non-cancer related pathways such as Hepatitis B, FoxO signaling pathway, PI3K-Akt signaling pathway, adherens junction, MAPK signaling pathway were also enriched.

**Table 4:** KEGG pathway analysis of mRNAs in the ceRNA network.

KEEG pathways	Count	*P* value*
*Cancer related*		
oas05206: MicroRNAs in cancer	31	3.44E−27
oas05200: Pathways in cancer	28	1.40E−14
oas05205: Proteoglycans in cancer	19	1.27E−11
oas05212: Pancreatic cancer	12	1.54E−10
oas05215: Prostate cancer	13	2.13E−10
oas05210: Colorectal cancer	11	1.82E−09
oas05218: Melanoma	10	9.46E−08
oas05230: Central carbon metabolism in cancer	9	5.69E−07
oas05214: Glioma	8	8.33E−06
oas05213: Endometrial cancer	7	2.56E−05
*Non-cancer related*		
oas05161: Hepatitis B	19	2.59E−14
oas04068: FoxO signaling pathway	18	1.02E−13
oas04151: PI3K-Akt signaling pathway	22	2.62E−10
oas04520: Adherens junction	11	5.30E−09
oas04010: MAPK signaling pathway	17	2.24E−08

* *P* value < .05 was considered as threshold values of significant difference.

### 3.6. Potential prognosis of LUAD specific lncRNAs in the ceRNA network

**Figure 3.**
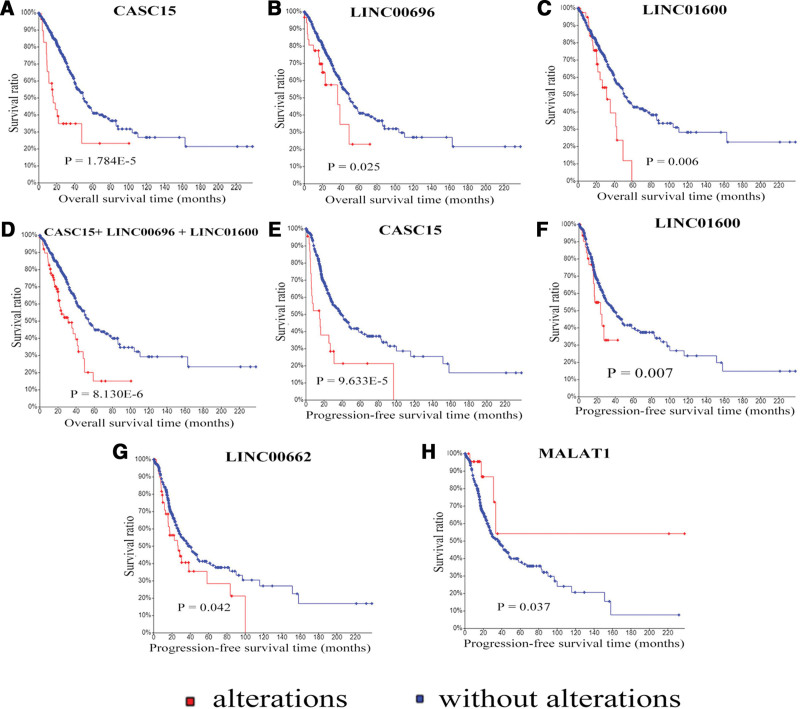
Kaplan–Meier analysis of prognosis related lncRNAs. (A–C) Kaplan–Meier curves of CASC15, LINC00696, and LINC01600 were associated with OS. (D) Kaplan–Meier curve of the combination of the above 3 lncRNAs. (E–H) Kaplan–Meier curves of CASC15, LINC00662, LINC01600, and MALAT1 were associated with recurrence. Red and blue dots indicate patient samples with or without genetic alterations, respectively. *P*-value indicates the significance of difference.

To identify the potential lncRNAs with prognostic characteristics, the expression levels of 24 LUAD specific lncRNAs in the ceRNA network were profiled using survival Kaplan–Meier estimate from cBioPortal. As a result, using Onco Query Language EXP > 2, 3 lncRNAs (CASC15, LINC00696, and LINC01600) were found to be significantly associated with overall survival (*P* < .05) (Fig. 3A–C). The combination of these 3 lncRNAs accounted for a total of 17.5% (90/ 517) cases and negatively correlated with OS (*P* = 8.130E − 6) (Fig. 3D). Recurrence is a major concern for cancer survivors. There were 187 cases of recurrence in this cohort. The results indicated that 4 lncRNAs (CASC15, LINC00662, LINC01600, and MALAT1) was significantly associated with recurrence (*P* < .05). Of those, 3 IncRNA, including CASC15, LINC00662, and LINC01600 were negatively correlated with progression-free survival (*P* < .05) (Fig. 3E–G). On the contrary, MALAT1 were positively correlated with progression-free survival time (*P* < .05) (Fig. 3H).

### 3.7. Clinical significance of CASC15 and LINC01600 in LUAD

**Figure 4.**
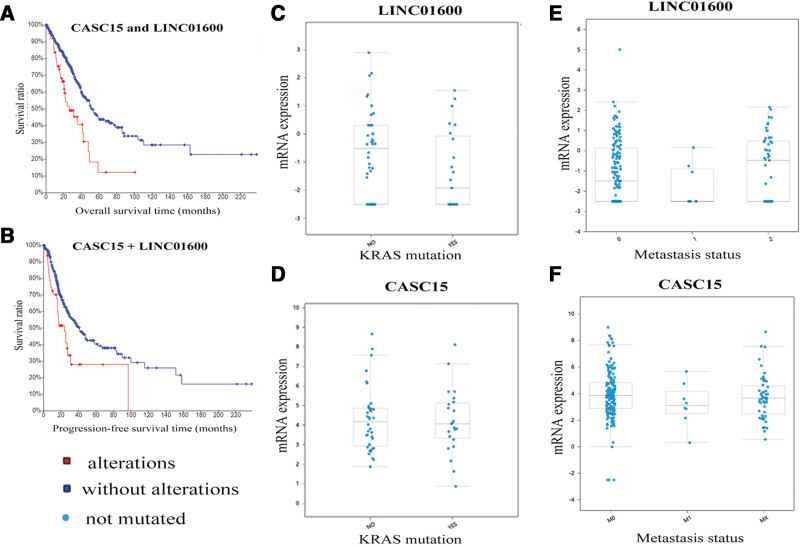
The prognostic characteristics of CASC15 and LINC01600 regarding OS, recurrence and other clinical features (lymph node metastasis and KRAS mutation). (A and B) The combination of CASC15 and LINC01600 dramatically negatively correlated with OS and recurrence (*P* < .05); (C–F) the expression of LINC01600 in cases with metastasis or KRAS mutation was significantly increased compared with cases without metastasis or KRAS mutation. However, there were no associations between CASC15 and metastasis status or KRAS mutation.

Based on above results, we noticed that 2 lncRNAs, CASC15, and LINC01600, performed the potential prognostic characteristics with LUAD regarding both OS and recurrence, suggesting they may exist as a synergic relationship. The combination of CASC15 and LINC01600 dramatically negatively correlated with OS and recurrence (*P* < .05) (Fig. 4A and B). To reveal the clinical significance of these 2 lncRNAs in LUAD, genetic alterations, as well as alterations to other clinical features, such as sex, age in diagnosis, tumor stage, metastatic status, lymph node metastasis and KRAS mutation, were investigated. Notably, LINC01600 was identified to be associated with KRAS mutation. As presented in Figure 4C, the expression of LINC01600 in cases without KRAS mutation was significantly increased compared with cases with KRAS mutation (*P* < .05). Furthermore, we found that the expression of LINC01600 in cases with metastasis (M = 1 or x) was significantly increased compared with cases without metastasis (M = 0) (*P* < .05) (Fig. 4E). However, there were no associations between CASC15 and metastasis status or KRAS mutation (Fig. 4D and F). Other clinical features, including sex, age in diagnosis, tumor stage, and lymph node metastasis, were evaluated separately, and no significant associations with CASC15 or LINC01600 were observed.

CBioPortal provided the probable co-occurrence of these 2 lncRNAs and there was a significant tendency towards co-occurrence between CASC15 and LINC01600 in LUSC (*P* = .021). The co-expressed genes of these 2 lncRNAs were also determined. As a result, 32 genes were revealed to be co-expressed with CASC15, and 125 genes were discovered to have a co-expressed relationship with LINC01600, based on the algorithm blow: Pearson correlation > 0.5 (Table S5, Supplemental Digital Content, http://links.lww.com/MD/K669). Go analysis indicated that biological and functional process of CASC15 and LINC01600 was similar. In the BP, both CASC15 and LINC01600 co-expressed genes were most enriched in extracellular exosome or space and in the CC analysis, both of them were most significantly involved in interferon-gamma-mediated signaling pathway, signal peptide processing and regulation of protein stability.

## 4. Discussion

Recently, aberrant expression of lncRNAs has been widely observed in various cancers and accumulative evidence indicates that lncRNAs emerge as key regulatory roles in tumorigenesis and tumor progression.^[28–31]^ Notably, in addition to directly interacted with mRNAs, lncRNAs can also function as endogenous miRNA sponges of a lncRNA–miRNA–mRNA based ceRNA network to indirectly regulate targeted mRNAs and several vital regulatory pathways, as well as potential therapeutic targets, may be revealed. Wang et al found that CircNT5E acted as a sponge of microRNA-422a to promote glioblastoma tumorigenesis.^[32]^ Mou et al described that lncRNA-ATB functioned as a competing endogenous RNA to promote YAP1 by sponging miR-590-5p in malignant melanoma.^[33]^ The above results elucidated the important roles of lncRNAs in tumorigenesis and tumor development as a part of ceRNA networks and suggested a potential strategy for the treatment of cancers.

To date, some efforts had been made to describe lncRNA profiles in LUAD and several dysregulated lncRNAs as ceRNAs were reported based on microarray analysis. Li et al revealed that MEG3 and MIAT regulate MAPK9 by interactions with miR-106 to involve in MAPK signaling pathways and LINC00115 might interact with miR-7 to regulate FGF2 to participate in pathways in cancer.^[34]^ These lncRNAs may be underlying therapeutic targets for LUAD functioning as ceRNAs for regulation of miRNA-mRNA. Sui et al found 21 lncRNAs in LUAD-related ceRNA network were aberrantly expressed with clinical features and 5 lncRNAs (BCRP3, LINC00472, CHIAP2, BMS1P20, and UNQ6494) positively correlated with OS.^[35]^ Zhao et al constructed a specific SVM (support vector machine) classifier based on the ceRNA network for diagnosis of LUAD and identified hsa-miR-96, hsa-miR-204, PGM5P2, SFTA1P, RGS20, RGS9BP, FGB and INA may serve as prognostic markers in clinical practice.^[27]^ However, studies on identification of the LUAD-related lncRNAs based on genome-wide RNA profiles and large sample size were still poorly described.

In the current study, we interrogated the LUAD dataset of TCGA with larger 517 tissue samples at cBioPortal with the latest updated lncRNAs and miRNAs database from HGNC. lncRNAs and miRNAs with highly genetic alterations were identified and subsequently selected to construct the regulated lncRNA-miRNA-mRNA based ceRNA networks. A total of 24 lncRNAs, 19 miRNAs and 142 mRNAs were involved in this network and GO and KEGG pathway analysis of targeted mRNAs was operated. The results of GO analysis indicated that most mRNAs in the ceRNA network were significantly enriched in biological or cellular process, cell components and kinase activities. Based on KEGG pathway analysis, in addition cancer-related pathways, some non-cancer related pathways, including FoxO signaling pathway, PI3K-Akt signaling pathway and MAPK signaling pathway were also enriched. Some studies had reported that these signaling pathways were involved in the initiation and progression of LUAD.^[36]^ Furthermore, among 24 lncRNAs in the network, several key lncRNAs were identified as LUAD prognosis-related. Three lncRNAs (CASC15, LINC00696, and LINC01600) were found to be negatively correlated with OS and 4 lncRNAs (CASC15, LINC00662, LINC01600, and MALAT1) was significantly associated with recurrence. Of those, CASC15, LINC00662, and LINC01600 were negatively correlated with recurrence, and conversely, MALAT1 were positively correlated with recurrence. LINC00696, LINC01600, and LINC00662 were novel identified LUAD prognosis-related lncRNAs and their functions remained elusive. MALAT1 was a well-known lncRNA, which can function as a ceRNA by sponging to miRNAs to indirectly regulate targeted mRNAs in various cancers. Luan et al found that MALAT1 acted as a ceRNA to promote malignant melanoma growth and metastasis by sponging miR-22.^[37]^ Zhang et al revealed that MALAT1 regulated the expression of Gli2 by miR-202 to strengthen gastric cancer progression.^[38]^ Tao et al discovered that miR-211 sponged MALAT1 to suppress tumor growth and progression through inhibiting PHF19 in ovarian carcinoma.^[39]^ Chang et al found that MALAT1 can function as a ceRNA to modulate STAT3 expression by absorbing miR-125b in oral squamous cell carcinoma (OSCC) and could be used as a novel therapeutic target in OSCC diagnosis and treatment.^[40]^ CASC15, cancer susceptibility candidate 15, originally named FLJ22536, was a long intergenic noncoding RNA (lincRNA) locus in chromosome 6p22.3.^[41]^ Lessard et al reported that CASC15 was involved in melanoma progression and phenotype switching, and was an independent predictor of recurrence for metastatic melanoma.^[42]^ Fernando et al found that CASC15 can regulate SOX4 expression in RUNX1-rearranged acute leukemia.^[43]^ Some other research described overexpression of CASC15 may promote tumor development and progression in gastric cancer and hepatocellular carcinoma, and was correlated with a poor prognosis.^[44,45]^ However, studies about how CASC15 functioned as a ceRNA were rare. Recently, Jing et al carried out a study and the results revealed that CASC15 promoted colon cancer growth and metastasis through the activation of the Wnt/β-catenin signaling pathway by acting as a sponge to suppress miR-4310 that targeted LGR5.^[46]^ This study suggested that CASC15 may be a therapeutic target for colon cancer treatment.

After systemic analysis of LUAD prognosis-related lncRNAs, interestingly, we found that CASC15 and LINC01600 both had the potential prognostic characteristics with LUAD regarding OS and recurrence, which drove us to explore their relationships and the correlation between them and other clinical features. We found that there were significant associations between LINC01600 and KRAS mutation or metastasis status, suggesting LINC01600 may be an oncogene and play a role in the KRAS related signaling pathways of LUAD. However, the associations between CASC15 and LINC01600 and other clinical features, including sex, age in diagnosis, tumor stage and lymph node metastasis, were observed. Moreover, bioinformatics analysis indicated that there was a significant tendency towards co-occurrence between CASC15 and LINC01600 in LUSC (*P* = .001). The results of Go analysis showed that the co-expressed genes of CASC15 and LINC01600 were similar, which were both enriched in extracellular exosome or space, and most significantly involved in interferon-gamma-mediated signaling pathway, signal peptide processing and regulation of protein stability. The similarity of co-expressed genes of CASC15 and LINC01600 may partly explain the reason why they had a significant tendency towards co-occurrence.

In this study, several notes should be mentioned. Firstly, for increasing the accuracy of ceRNA network prediction, we only recruited the cancer specific lncRNAs and miRNAs that had alterations more than 5% and were annotated by HGNC. The relationships between lncRNA and miRNA, and miRNA and mRNA were predicted by experiment-supported algorithms or databases such as starBase and miRTarBase. These measurements guaranteed that the relationships identified would be reliable not only in silico situations but also by experimental-supported evidences. However, the limitation of the constructed ceRNA network and LUAD prognosis-related lncRNAs were still obvious due to the lack of experiment validation. Further experiments, such as reporter assay, western blot, or qPCR methods were mandatory to confirm these results. Moreover, since TCGA was a public and accessible cancer database, it was inevitable to come across the situation that results were obtained from different studies based on the same database. For example, Zhao also used tumor sample data from TCGA to construct a LUAD related miRNA–lncRNA–mRNA network and identified molecular markers with diagnostic and prognostic value for LUAD.^[27]^ However, there were still some differences between us. Firstly, the numbers and names of molecules in the constructed ceRNA network were different: there were 6 lncRNAs, 25 miRNAs and 126 mRNAs in Zhao work; in our study, the numbers of lncRNAs, miRNAs, and mRNAs were 24, 21 and 142, respectively. Secondly, 2 lncRNAs, 2 miRNAs, and 4 mRNAs were identified to be prognosis related in Zhao work while we focused on lncRNAs. Molecular markers in Zhao study were associated with OS; however, we found several lncRNAs performed the potential prognostic characteristics with LUAD regarding not only OS, but also recurrence, which revealed their relationships and the correlation between them and other clinical features.

In conclusion, in the present study, we draw aberrant expression profiles of LUAD-related lncRNAs and miRNAs from hundreds of candidate lncRNAs detected from large samples size in TCGA database and constructed the lncRNA–miRNA–mRNA ceRNA network to clarify the unknown ceRNA regulatory network in LUAD. Some key lncRNAs were subsequently identified as LUAD prognosis-related, and of those, CASC15 and LINC01600 both performed the potential prognostic characteristics with LUAD regarding OS and recurrence. Comprehensive analysis indicated that the expression of LINC01600 was significantly associated with KRAS mutation and lymph node metastasis, and CASC15 and LINC01600 were significantly tended towards co-occurrence, which may be due to the similarity of genes co-expressed by these 2 lncRNAs. Our findings provided novel insight into better understanding of the lncRNA–miRNA–mRNA based ceRNA network in LUAD and potential biomarkers for prognosis.

## Author contributions

**Conceptualization:** Fangbin Zhou.

**Data curation:** Fangbin Zhou.

**Formal analysis:** Fangbin Zhou.

**Funding acquisition:** Fangbin Zhou.

**Investigation:** Fangbin Zhou.

**Methodology:** Fangbin Zhou.

**Project administration:** Fangbin Zhou.

**Resources:** Fangbin Zhou.

**Software:** Fangbin Zhou.

**Supervision:** Fangbin Zhou.

**Validation:** Fangbin Zhou.

**Visualization:** Fangbin Zhou.

**Writing – original draft:** Fangbin Zhou.

**Writing – review & editing:** Fangbin Zhou.

## Supplementary Material










